# Rupture of Caseous Calcification of the Mitral Annulus: Pathophysiology, Diagnosis and Treatment

**DOI:** 10.3390/diagnostics16050778

**Published:** 2026-03-05

**Authors:** Aureliano Ruggio, Antonietta Belmusto, Gabriella Locorotondo, Eleonora Ruscio, Francesca Graziani, Antonella Lombardo, Gaetano Antonio Lanza, Francesco Burzotta

**Affiliations:** 1 Department of Cardiovascular Sciences, Fondazione Policlinico Universitario A.Gemelli, IRCCS, 00168 Rome, Italy; aureliano.ruggio@policlinicogemelli.it (A.R.);; 2 Department of Cardiovascular Sciences-CUORE, Catholic University of the Sacred Heart, 00168 Rome, Italy

**Keywords:** rupture, caseous necrosis, mitral annulus calcification, complication, multimodality imaging, diagnosis, management

## Abstract

Caseous calcification of the mitral annulus (CCMA) is a liquefactive necrosis of mitral annular calcification (MAC). CCMA is rare and usually asymptomatic, has a benign course, and, when incidentally found, can be misdiagnosed as a thrombus, abscess, cardiac tumor or vegetation. Although rarely, CCMA may complicate with rupture, which can lead to ventricular-atrial fistulization, pseudoaneurysm, severe mitral regurgitation (with possible heart failure and atrial fibrillation) and systemic embolism of caseous material (with cerebral ischemic events). A significant increase in CCMA dimensions and an infectious involvement of liquefactive necrosis make CCMA prone to rupture. To date, only case reports and some case series have been published on CCMA, without focusing on the pathophysiological mechanisms responsible for rupture, nor recommendations for prevention and management. However, despite general concerns about surgical treatment of CCMA because of high perioperative risks, most published cases actually underwent successful cardiac surgery. In the present review, we conducted a systematic review of the studies published in the medical literature up to March 2025, reporting cases of CCMA and its complications, as identified through the PubMed database. We analyzed clinical and biological risk factors for CCMA rupture and its diagnostic criteria, focusing on imaging features differentiating mitral annular calcification from uncomplicated CCMA and ruptured CCMA. To this regard, we focused on the key role of multimodality imaging in the achievement of the correct diagnosis. Finally, we propose a management strategy for CCMA, with the aim to fill a gap in this field in the current literature.

## 1. Introduction

Calcification of the aortic and mitral valves affects a substantial proportion of patients and can be due to a wide spectrum of pathophysiological mechanisms, spreading from atherosclerosis to inflammation and dystrophic degeneration. Mitral annular calcification (MAC) is a chronic degenerative process involving the degeneration and progressive calcium deposition within the fibrous skeleton of the mitral valve (MV) annulus, which is associated with advanced age, particularly in women, and various comorbidities, such as hypertension and chronic kidney disease [1].

Caseous calcification of the mitral annulus (CCMA) is a rare variant of MAC, consisting in the occurrence of a liquefactive necrosis within a severely calcified mitral annulus. Since most cardiologists are unfamiliar with CCMA, it is commonly misdiagnosed as an abscess, tumor or infective vegetation on the mitral valve [2,3]. Several pathophysiological mechanisms have been identified that can cause MAC to evolve into CCMA. Moreover, under specific clinical conditions, CCMA may disrupt, leading to fistulization, MV dysfunction, small erosion and systemic embolization of the caseous material, as well as pseudoaneurysm formation. Thrombosis, cerebral ischemia and severe mitral regurgitation are the most frequent complications of CCMA rupture [2].

Because of its rarity, even experts in cardiac imaging might be unfamiliar with such catastrophic complication, which can result in misdiagnosis and even inappropriate management. Nonetheless, as it may represent a dramatic event, rupture of CCMA needs to be adequately recognized and treated.

Although CCMA has previously been described in the literature, the mechanisms and consequences of CCMA rupture have not hitherto been systematically analyzed and highlighted.

In the present article, we focalize our attention on current epidemiological, pathophysiological and clinical knowledge of complicated CCMA, including potential risk factors for CCMA rupture, and highlight the potential role of multimodality imaging in the diagnostic approach of complicated CCMA, including identification of imaging features potentially requiring intervention or a careful follow-up and surveillance, thus trying to fill some relevant gaps in this filed. Finally, in the absence of a standardized treatment strategy in the literature, we also try to propose a flow-chart on the management of both uncomplicated and complicated CCMA.

To these scopes, we conducted a systematic search of studies published up to March 2025 in the medical literature, across the PubMed database, by using as keywords “caseous calcification of mitral annulus”, “mitral annulus caseous necrosis”, “mitral annular calcification” and “rupture”, “complications”, “endocarditis”, “fistulization”, “heart failure”, “mitral regurgitation”, “embolism” and “multi-modality imaging”.

### 1.1. Anatomy and Epidemiology of Mitral Annular Calcification

The MV annulus is a saddle-shaped structure. When occurring, MAC mainly involves the posterior portion of the annulus, due to anatomical and functional reasons. While the anterior mitral annulus is structurally reinforced by dense connective tissue and closely integrated with the cardiac fibrous skeleton (particularly the aortic annulus), the posterior annulus has, indeed, a weaker C-shaped structure, extending from the left to the right fibrous trigone of the MV and only loosely connected to the posterior left atrial and left ventricular myocardium. As a consequence, the posterior mitral annulus undergoes greater excursions than the anterior one throughout the cardiac cycle and is, therefore, more susceptible to mechanical stress, which makes it more vulnerable to degenerative changes and calcium deposition [4].

Moreover, despite the name, MAC is a condition that frequently extends beyond the annulus, involving the mitral leaflets, the papillary muscles, the base of the left ventricle, the chordae tendineae and the mitral-aortic intervalvular fibrosa [5]. In a case series of 68 patients with extensive calcification of the MV annulus and severe MV insufficiency undergoing surgery, the intra-operative examination of the annular extension of the calcification process revealed that the calcification process remained localized to the annulus in 77% of the cases, involving more than one third of the annulus in 88% of the patients, the posterior annulus only in 10.5% and the whole annulus in 1.5%, whereas, it extended to the myocardial wall in 12% of the patients and to the papillary muscles in 4.5% [6].

While MAC is a common finding, with a prevalence of up to 23% in elderly patients, the prevalence of CCMA is less than 0.1% in the general population. According to large autopsy series in the literature, however, the prevalence of CCMA among patients with MAC may range from 0.6 up to 2.7%, thus indicating that this condition may be underdiagnosed [2,7,8]. Notably, the rupture of CCMA is a rare complication, described in the literature only in isolated case reports and small case series [Table 1].

### 1.2. Anatomical Features and Autoptic Findings

During surgical exploration, CCMA presents as an intramyocardial rounded mass localized in the region of the mitral annulus, encapsulated within a rigid, non-collapsible, heavily calcified envelope. Upon incision of the lesion, a “toothpaste-like” caseous material is extruded. CCMA most commonly involves the posterior annulus, with only a few cases documented involving the anterior annulus only. In a study by Harpaz et al., among 19 patients with CCMA, only one exhibited an involvement of both anterior and posterior annulus [7]. Likewise, Deluca et al. reported that only 2 out of 14 cases demonstrated CCMA affecting both annuli, whereas all other lesions were confined to the posterior annulus [22]. The reason underlying the preferential localization to the posterior annulus of CCMA may reside in the higher prevalence of MAC in the posterior region.

The microscopic examination of the caseous material reveals a central area of amorphous, eosinophilic, acellular material, also containing calcium, cholesterol and fatty acids, surrounded by macrophages and lymphocytes, with peripheral regions showing multiple calcifications and zones of necrosis [23].

In this regard, Tanaka et al. have reported an interesting case of an 85-year-old woman with CCMA diagnosed by a multi-modality imaging approach. By integrating the information derived from different imaging modalities, the authors speculated that the liquefaction of the internal part of MAC may have caused the formation of a mass containing high-protein and hemorrhagic material. Indeed, the pathological and gross findings demonstrated that CCMA included protein and hemorrhagic components, together with calcification and necrotic areas [15].

When CCMA evolves with rupture, no fistulous break can often clearly be seen, even at surgical inspection; its presence, however, can be suspected by the detection of amorphous caseous material attached to the chordae tendineae and papillary muscles. In this regard, Fujiwara et al. reported three cases of CCMA with surgically confirmed spontaneous fistula formation [16]. Particularly, in a case of a 61-year-old man with an incidental finding of a mobile mass on the posterior MV leaflet, carrying a high risk of embolism, the intraoperative examination showed that the wall of the mass was hard, but, after opening, revealed a white, milky, “toothpaste-like’’ material, typical of CCMA. On pathological examination, the mass contained a central region of amorphous eosinophilic acellular material, surrounded by macrophages and lymphocytes, with multiple calcifications and necrotic zones at the periphery. The mobile string-like structure seen on echocardiography was found to be a calcified structure entwined within, but separate from, the chordae tendineae and attached to the papillary muscle. Thus, a fistulous communication between the CCMA mass and the left ventricle was suspected, and the mobile calcified mass could potentially be a source of embolization [16].

## 2. Pathophysiology of CCMA Formation and Progression

Both MAC and CCMA share similar risk factors with atherosclerosis, such as age, female sex, hypertension, smoking habit and family history of myocardial infarction [24]. Furthermore, conditions associated with increased mitral valve closing pressure (i.e., hypertension, hypertrophic cardiomyopathy or aortic valve stenosis) can exert excessive annular tension, thus promoting dystrophic calcification of the annulus [24]. Patients with congenital connective tissue disorders (such as Marfan syndrome) and mitral valve prolapse also share an increased stress on the MV annulus, due to redundant, hypermobile leaflets, leading to accelerated tissue degeneration [6,7,25]. Moreover, systemic conditions predisposing to calciphylaxis, such as chronic kidney disease or other metabolic disorders that result in abnormal calcium-phosphorus metabolism, also favor metastatic calcification of the mitral annulus, thus predisposing to CCMA [16,24,26].

Finally, MAC and CCMA exhibit a higher prevalence in post-menopausal women, suggesting a peculiar association between age-related osteoporosis and ectopic calcium deposits. Indeed, MAC prevalence has been shown to be inversely related to bone mineral content. Furthermore, among women, the frequency of osteoporosis was found to be higher in patients with MAC than in patients without MAC. A possible explanation for this finding is that bone decalcification is responsible for high serum levels of ionized calcium and consequent ectopic calcium deposition on mitral annulus [27,28].

The exact pathophysiologic mechanisms involved in the progression from MAC to CCMA are not completely understood. MAC, however, rarely evolves into caseous degeneration of its inner core material. A lot of evidence suggests that CCMA is not simply a degenerative accumulation of inert calcium substrates, but, rather, it is sustained by a dynamic, mainly inflammatory, process culminating into a rupture of the calcified mass. CCMA and atherosclerotic lesions share common histologic evidence of inflammation, characterized by lipid and macrophages infiltration [22]. Particularly, CCMA is associated with blood inflammatory markers, such as C-reactive protein and interleukin-6 [24], and is characterized by increased local calcification activity and inflammation [29]: it is plausible that a self-perpetuating cycle of calcification and inflammation within MAC, virtually driven by local inflammation and mechanical stress, may eventually lead to CCMA.

The same factors involved in CCMA formation, such as chronic kidney disease, diabetes, hypertension, dyslipidemia and increased mechanical stress, are thought to be related to its progression and eventually to its rupture.

On the other hand, three cases in the literature have reported a regression of CCMA, which was found to undergo spontaneous shrinkage with a reduction of its dimensions, or even revert to MAC [30,31,32]. In the two cases in whom CCMA showed a spontaneous shrinkage at 9 months [31] and 1 year [30] after the initial diagnosis, the authors hypothesized that a central liquefaction and dissolution of the material through the rupture of the external wall occurred, without any evident clinical consequences. In the third case, a hemodialysis regimen with a low calcium concentration was applied in a patient with chronic kidney disease, which resulted in the echocardiographic evidence of a regression to MAC of CCMA (reduced echo-dense mass without the echo-lucent area) three months later [32]. Thus, the reduction of the mass observed after changing hemodialysis treatment with low calcium concentration led the authors to suppose that the acute change in serum calcium level might have contributed to the regression of CCMA and reduction of MAC.

The paucity of available clinical and anatomical data, however, is not sufficient to clarify the exact pathophysiologic mechanisms responsible for spontaneous CCMA regression, but again, they highlight the dynamic nature of CCMA. We could speculate that the “dynamic” nature of calcium degeneration could have a potential role for such a favorable outcome. Further studies on larger cohorts of patients are needed to better understand this complex multifactorial process.

### Risk Factors for CCMA Rupture

The exact incidence of CCMA rupture and the time interval between CCMA diagnosis and this catastrophic complication are unknown. In the absence of large longitudinal cohort studies exploring the evolution over time of CCMA and addressing the rate of CCMA rupture, we can only try to derive this information by published case report. By performing a systematic literature search across the PubMed database, 196 papers can be found about CCMA. Among them, only 13 (which are summarized in Table 1) clearly reported rupture of CCMA. Thus, rupture is described in 7% of papers reporting CCMA. The time interval between MAC or CCMA diagnosis and CCMA rupture was extremely heterogeneous, ranging from a minimum of 1 months [9] to a maximum of 8 years [19].

Although the exact mechanisms responsible for CCMA rupture cannot be determined with certainty, some common features seem to be present in patients affected by rupture of CCMA [Table 1], which can be distinguished in local and systemic factors.

***Calcification size and mobility***: These findings probably represent the most frequent characteristics associated with rupture: indeed, ruptured CCMA showed greater dimensions and mobility than simple MAC and uncomplicated CCMA [Table 1]. Furthermore, CCMA exposition to a higher hemodynamic stress may also contribute to rupture.

***Diseases of the myocardium***: CCMA can be prone to enlarge within the affected interstitium and, when ruptured, lead to pseudoaneurysm formation; indeed, rupture of CCMA has been described in both hypertrophic cardiomyopathy [19] and amyloid infiltrative cardiomyopathy [20].

***Infective endocarditis and sepsis:*** Systemic infections have been frequently described in patients showing rupture of CCMA. The liquefactive necrosis within CCMA may represent a vulnerable lesion to bacteria (especially *Staphylococcus* spp.), resulting in infective endocarditis [33]. *Staphylococcus* spp. may infect CCMA because of their specific affinity for osteoblasts. CCMA may act as A nidus for infection, especially by *S. aureus*. Differences in mechanism of attachment between *S. aureus* and *Streptococci* may account for the observed difference in frequency of attachment of vegetations to MAC [10]. In an elegant observational study by Eicher JC et al., MAC appeared to be an underestimated predisposing factor for a particularly severe type of bacterial endocarditis. Among 62 patients with infective endocarditis of a native MV diagnosed with multiplane TOE, the group of patients with vegetations originating from MAC differed significantly from that with a classic leaflet endocarditis with regard to a higher prevalence of diabetes mellitus and cancers, a more severe initial clinical presentation (febrile coma or meningoencephalitis in 53% of cases), echocardiographic features (significantly greater vegetations, presence of calcium-dense echoes within the vegetation, high rate of ring abscess and para-annular ventricular-atrial leakage) and poorer clinical outcome (53% of in-hospital mortality) [11].

## 3. Clinical Manifestations of CCMA Rupture

While CCMA is generally an incidental asymptomatic finding during routine transthoracic echocardiography, its rupture may have severe and life-threatening consequences.

1. ***MV dysfunction and heart failure***. Even when uncomplicated, CCMA may cause a structural deformation of the MV apparatus and loss of physiological mitral annular dynamics, due to annular calcium infiltration and restricted leaflet mobility, leading to both MV regurgitation [12] and stenosis [34]. According to case series in the literature, the occurrence of MV regurgitation ranges from 63% to 100% and mitral stenosis from 14 to 16% [7,22]. When CCMA complicates with rupture, disruption of the mitral annulus creates a direct communication between the left ventricle and the left atrium (i.e., ventricular-atrial fistulization). An increase in the systolic pressure within the left ventricle may in fact determine the rupture of the fragile liquefactive mitral annulus and cause the mitral regurgitation jet to pass through the disrupted annulus. In these cases, perivalvular jets contribute to determine a severe mitral regurgitation, possibly resulting in pulmonary oedema, reduced anterograde systolic output and heart failure.

2. ***Arrhythmias.*** Even when uncomplicated, CCMA is associated with an increased risk of arrhythmias, including both brady-arrhythmias and tachy-arrhythmias. As described in isolated case reports, a direct extension of calcific infiltration into the region of the atrioventricular node and His bundle can result in conduction disturbances, potentially manifesting as various degrees of atrioventricular block and bundle branch block, often requiring a permanent pacemaker [35,36]. Moreover, the altered mitral annulus dynamics may lead to chronic atrial remodeling, predisposing to the development of atrial fibrillation, with a variable frequency (10.5–35.7%) [7,22]. When CCMA complicates with rupture, a rapid great increase in the left atrial pressure may further facilitate the development of tachyarrhythmias, such as atrial fibrillation. The management of atrial fibrillation can be more challenging in these cases, as CCMA rupture may represent a perpetuating mechanism of atrial impairment. Moreover, in case of atrial fibrillation complicating a CCMA rupture, it can become difficult to establish the exact cause of any potential systemic embolism (e.g., embolism from the caseous material or an intra-atrial thrombus).

3. ***Systemic embolization.*** A possible adverse evolution of CCMA is by itself systemic embolization, which can mainly cause ischemic stroke [37], due to the release of calcific debris into the bloodstream. In 2016, Dietl et al. reported a 19.2% incidence of embolic events in CCMA, significantly higher than the 11.8% reported in patients with MAC, challenging the notion that CCMA is a uniformly benign entity [38]. Spontaneous fistulization of the caseous necrosis and direct release of caseous material into the left atrium or ventricle has also been described in the literature [9].

However, the actual association between these clinical manifestations and CCMA should be considered with caution. Particularly, the occurrence of both tachy-and brady-arrhythmias could also be related to age and the degenerative changes in the conduction system might take place independently of CCMA. At the same time, as far as systemic embolization is concerned, CCMA should be considered as the cause of an ischemic stroke only after an extensive diagnostic work-up has excluded other possible common causes of the stroke.

## 4. Multimodality Imaging

Multimodality imaging ensures an accurate diagnosis, especially in difficult cases, and enables a targeted therapeutic strategy and follow-up. Although a systematic imaging protocol for CCMA has not been recommended yet, a discussion about potentials and limits of each imaging modality may inform a general guide. Echocardiography certainly represents the first-line imaging modality for the assessment of any structural and functional abnormality of the mitral annulus. Particularly, when the origin and entity of MR cannot be exactly identified or when systemic embolization is suspected, in absence of clear evidence of caseous material leakage at the transthoracic approach, or in case of a suspected infectious involvement of CCMA, transesophageal echocardiography is essential to better characterize the anatomy and function of the mitral valve, and then to plan treatment. Secondarily, imaging modalities such as computed tomography (CT), cardiac magnetic resonance (CMR) and positron emission tomography (PET), can provide valuable information enabling differential diagnosis with other potential intracardiac masses, such as thrombosis, abscesses and tumors. The choice among these second-level imaging modalities greatly relies on the specific question that needs to be addressed. Indeed, while all of them are equally valuable in the differential diagnosis between thrombosis and abscesses or tumors, some peculiarities may make one imaging modality more useful than another one for some specific questions. For instance, the capability of CT to clearly detect calcified tissues allows to precisely evaluate the extent of calcification within mitral annulus or myocardium. On the other hand, the possibility of CMR to provide a tissue characterization by multiple imaging sequences allows to clearly demonstrate inflammatory involvement of CCMA or to confirm its caseous nature. Moreover, cardiac uptake of 18-labeled fluorodeoxy-glucose (18F-FDG) in the context of sepsis may confirm CCMA involvement in the systemic inflammatory process.

Some imaging features, able to differentiate MAC from uncomplicated CCMA and ruptured CCMA, need to be recognized (Table 2).

**Figure 1:**
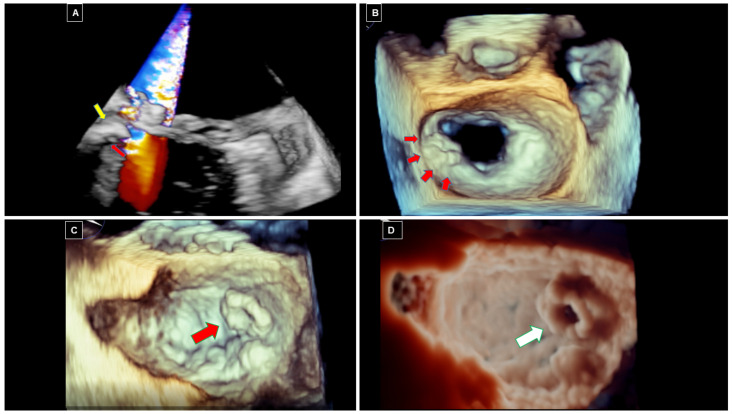
Three-dimensional transesophageal echocardiography details of ruptured CCMA. (**A**) shows color-Doppler assessment of ventricular-atrial fistulization and relationship with ruptured medial annulus and commissure. (**B**,**C**) show volume rendering surgical views of mitral valve with ruptured CCMA at lateral (**B**) and medial (**C**) portion of mitral annulus in two different patients. (**D**) shows the same image of (**C**), but highlighted by transilluminating mode, clearly demonstrating a hole in the lesion appearing as a crater.

At transthoracic and transesophageal echocardiography (TTE and TOE, respectively), both MAC and CCMA appear as a large, rounded, echo-dense mass located in the peri-annular mitral region [Figure 1].

**Figure 2:**
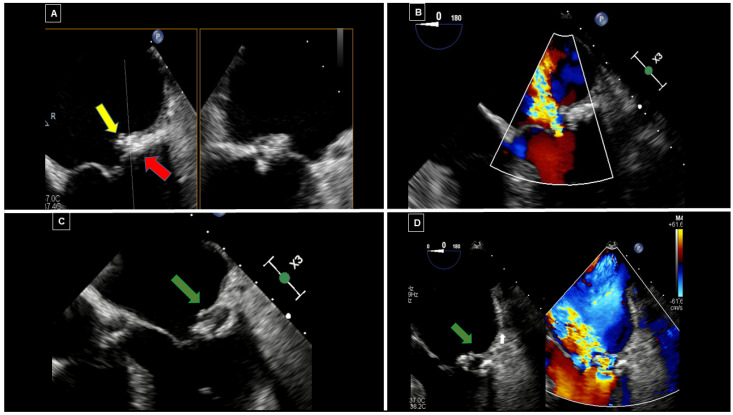
Evolution of MAC towards CCMA with fistulzation at TOE. Transesophageal echocardiography (TOE) black and white (**A**,**C**) and color-Doppler mode (**B**,**D**) images at mid-esophageal 0° view, acquired at first evaluation (**A**,**B**) and after 2 weeks (**C**,**D**) in a 77-year-old woman with infective endocarditis of the mitral valve due to Methicillin-susceptible Staphylococcus Aureus (MSSA) with cerebral embolization.

MAC can present as either circumferential or focal and typically shows posterior acoustic shadowing and lacks a central echo-lucent core. In contrast, CCMA presents central echo-lucent areas suggestive of liquefaction, without any acoustic shadowing [Figure 2]. It may appear semilunar, located in the posterior MV annulus at the junction between the left atrium and left ventricle, partially involving the body of the posterior mitral leaflet [red arrows in Figure 2]. When infected, a superimposed softer isoechoic and mobile mass, suggestive of a vegetation, can be found [yellow arrow in Figure 2]. If CCMA evolves towards rupture, a large central anechoic area, suggestive of liquefaction, due to an infective process [green arrow in Figure 2], can be associated with a ventricular-atrial fistulization through CCMA. Notably, color-Doppler evaluation is essential to detect complications. Thus, MV dysfunction associated with CCMA is usually characterized by a mitral regurgitation jet passing through the leaflet coaptation [Figure 2B]. However, when rupture and ventricular-atrial fistulization occur, a blood signal flowing to the central necrotic areas and connecting two cardiac chambers is usually seen [14,23] [Figure 2], with a severe MV regurgitant jet crossing the ruptured lesion [white arrow in Figure 2].

**Figure 3:**
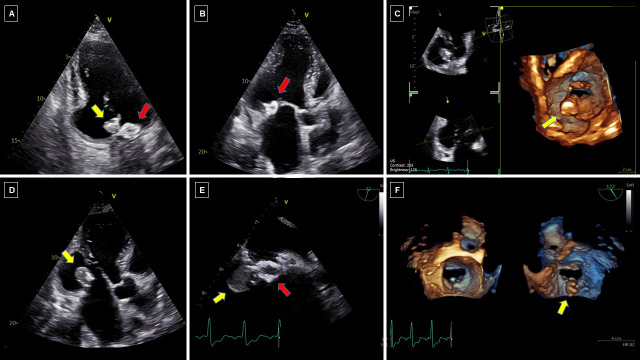
Echocardiography of ruptured CCMA with leaking caseous material. Two-dimensional (**A**,**B**,**D**,**E**) and three-dimensional (**C**,**F**) transthoracic (**A**–**D**) and transesophageal (**E**,**F**) images in a 69-year-old man with previous inferolateral ST-segment elevation myocardial infarction treated by percutaneous coronary intervention with stent implantation on left circumflex artery, showing an intracardiac mass suspected for leaking caseous material.

In ruptured CCMA, TOE allows a better visualization of the excavation with frayed and fluctuating edges on the atrial side, in case of superimposed infective endocarditis [14], or of soft echo-dense tissue consisting in caseous material coming out from the lesion [Figure 3]. When caseous material leaks from CCMA, the large, rounded, echo-dense mass [red arrow in Figure 3] located at the level of the mitral annulus, with a hyperechoic border and a central echo-lucent area, suggestive of liquefaction, appears in continuity with a larger rounded hypoechoic mass [yellow arrow in Figure 3], apparently originating from the inner anechoic core.

**Figure 4:**
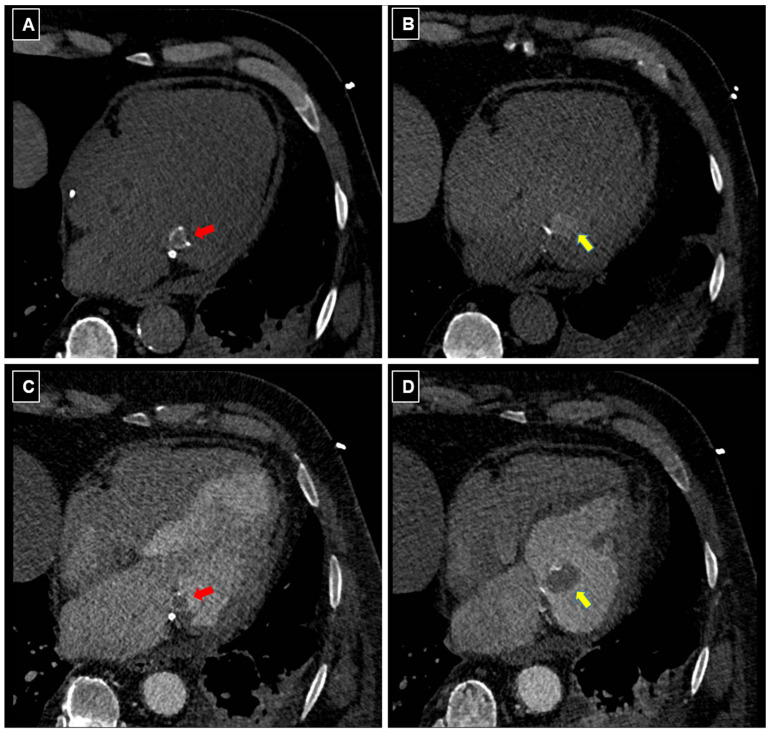
Computed tomography (CT) features of complicated CCMA. Baseline (**A**,**B**) and following contrast enhancement (**C**,**D**) trans-axial CT images.

At CT imaging, CCMA appears as an ovoid mass in the peri-annular region of the mitral valve and may show an average density of 40 HU [Figure 4]. The hypodense ovoid mass shows a central core of homogeneous or heterogeneous density (calcified, lipid-rich and fibrous content), consistent with liquefactive necrosis, and a ring-like peripheral calcification [red arrows in Figure 4]. When CCMA complicates with rupture, the protrusion of caseous material from its central core can be seen as an additional intracardiac mass in continuity with the CCMA [yellow arrows in Figure 4], presenting as slightly hyperdense at baseline and hypodense after contrast administration.

Following contrast administration, uncomplicated CCMA typically demonstrates no enhancement, a feature that helps to distinguish it from other cardiac masses, such as tumors [19,39]. Instead, in ruptured CCMA, the contrast medium typically fills the space between the posterior mitral valve and basal lateral myocardial wall with a peripheral rim of calcification. Any attached mass showing heterogeneous density like the CCMA core is suggestive of caseous material coming out [Figure 4].

**Figure 5:**
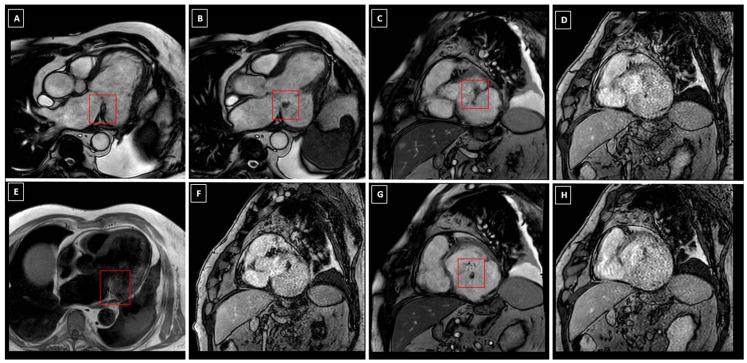
Cardiac magnetic resonance (CMR) features of complicated CCMA. (**A**,**B**) show bright-blood balanced steady-state free precession (b-SSFP) sequence in two different slices in three-chamber view. (**C**,**G**) display b-SSFP sequence in short axis view, at the mitral annulus level (**C**) and at the left ventricular basal level (**G**); corresponding slices acquired by inversion recovery gradient-echo (IR-GRE) late gadolinium enhancement (LGE) sequence with long inversion time (600 ms) to null thrombus signal are showed in (**D**,**H**), respectively. (**E**) shows black-blood T1-weighted fast spin echo (FSE) sequence, while (**F**) displays IR-GRE LGE sequence acquired with standard inversion time of 250 ms. Red frame indicates ruptured caseous calcification of the mitral annulus with leaking caseous material.

On CMR, T1- and T2-weighted sequences are useful in assessing the size and extent of the cardiac mass, due to their different signal intensity relative to the myocardium. MAC appears hypointense in T1-weighted, T2-weighted, gradient echo and steady-state free precession sequences. Conversely, in uncomplicated CCMA, the signal intensity on T1-weighted sequences may vary depending on the degree of calcification, protein and hemorrhage content, thus explaining the described variability in signal intensity [40,41] [Figure 5].

T1-weighted fast spin or turbo spin echo sequences typically reveal a lesion with a hyperintense core and a hypointense rim, distinct from the adjacent myocardium and posterior MV. In contrast, T2-weighted turbo spin echo and short tau inversion recovery (STIR) sequences show a centrally hypointense mass with surrounding ring-like hyperintensity. However, Monti et al. reported two cases of CCMA showing low signal in both T1-weighted and T2-weighted sequences [40]. On the other hand, Di Bella et al. found a hyperintense center and a hypointense rim in T1-weighted sequences and low signal in T2-STIR sequences [41]. As almost all other cardiac masses are hyper-intense on T2-weighted sequences, hypointensity on T2-weighted imaging is a useful finding for the diagnosis of CCMA. Post-contrast and perfusion sequences demonstrate the absence of central perfusion [42] and no significant late gadolinium enhancement at the core of the CCMA mass. The absence of vascularization distinguishes CCMA from benign or malign tumors, but a thin peripheral ring of late gadolinium enhancement can be determined by the CCMA capsule, although CMR does not disclose calcified areas as precisely as CT does [43]. Ruptured CCMA is characterized by hypointense rim on T1-weighted and T2-weighted sequences, with black-blood filled cavity in spin-echo sequences and bright-blood filled cavity in gradient-echo and steady-state free precession sequences. Late gadolinium enhancement sequences with long inversion recovery time, specifically chosen to null thrombus signal, may help in the differential diagnosis between thrombus (which remains hypointense) and caseous material (which shows hyperintensity like left ventricular cavity and walls).

Finally, positron emission tomography (PET) is a functional imaging technique that measures tissue metabolic activity using radiopharmaceuticals labeled with positron-emitting isotopes. CCMA is considered a dynamic pathologic process, reflecting ongoing metabolic, inflammatory and structural changes within the lesion. Therefore, depending on the choice of the radiotracer, PET allows for the assessment of the mass calcification activity and the degree of inflammation. The uptake 18F-FDG within the mitral annulus may correlate with active inflammation, being caused by its accumulation within macrophages engaged in the lesion [29]. PET utility is also well recognized in cases of suspected infective endocarditis, particularly for detecting active infectious foci involving CCMA [44]. On the other hand, an uptake of gallium-citrate 67 (^67^Ga-citrate) and technetium Tc 99m methylene diphosphonate (99mTc-MDP) can be observed in ongoing heterotopic calcifications, suggesting exuberant osteogenic activity involving mesenchymal metaplasia and both osteoblastic and osteoclastic processes. In contrast, the lack of uptake of these radiotracers suggests that the lesions are metabolically inactive, with stable forms of calcification, such as MAC [32]. When ruptured, CCMA occurs in the context of sepsis, the intense and diffuse myocardial uptake, suggestive of widespread inflammatory and metabolic activation that may prevent an adequate evaluation of the mitral annulus uptake (Table 2).

In summary, a systematic imaging protocol should start with a careful echocardiographic evaluation. This can be limited to a transthoracic approach, which may accurately identify the presence and extent of CCMA, as well as its overt rupture, but also requires a transesophageal approach in cases showing high-risk factors, which may reveal small erosions, superimposed infective vegetations or protruding caseous material. When the differential diagnosis between thrombus and caseous material is uncertain, CMR may provide tissue characterization. Alternatively, when CMR with gadolinium contrast agent is contraindicated or not tolerated in poor breath-holder patients, a detailed evaluation of CCMA and its complication can be obtained by CT. CT is also useful to plan surgical or percutaneous treatment, by giving information about the circumferential distribution or intramyocardial extent of annular calcium. PET, on the other hand, is less frequently performed than CMR and CT.

## 5. Management of Ruptured CCMA

Currently, there is no standardized management protocol for CCMA. Treatment is typically tailored to the individual patient, based on clinical presentation and imaging profile, as well as morphological characteristics of the mass. Cardiac surgery would represent the treatment of choice for removing CCMA, but most patients are denied due to perceived high risk of intraoperative complications, such as rupture of the mitral annulus or myocardium beneath the lesion. Nonetheless, the possibility that CCMA could regress to MAC [7,22,30] led some authors to state that, in the absence of clinical manifestations, close surveillance without treatment seems to be a good alternative option [7,22].

***Uncomplicated CCMA and absence of high-risk features.*** In uncomplicated CCMA and in the absence of high-risk features, a conservative management can be justified, as long as a watchful waiting approach can be undertaken. When a conservative approach is chosen, regular echocardiographic follow-up is generally considered the most prudent strategy [26], with regular clinical and echocardiographic follow-up intervals of 6–12 months, performed in the setting of a Heart Valve Clinic, in agreement with the latest ESC guidelines for the management of moderate-to-severe asymptomatic primary mitral regurgitation [45].

However, technical skills and expertise of surgeons should also be taken into account in the therapeutic decisions by the heart team, which may suggest an interventional approach in cases suggesting increased risk of CCMA complications, such as a rapidly expanding CCMA, as seen in dialysis patients [18], or in cases of increased infectious risk.

***Complicated CCMA.*** In complicated CCMA, that is CCMA associated with MV dysfunction, embolic manifestations, infective endocarditis, fistulization or when the possibility of a tumor cannot be ruled out [2,46], surgical intervention (i.e., MV replacement or repair) is indicated and has been performed successfully [Table 1]. No standardized surgical technique is described or recommended. As a surgical approach, some authors indicated a debridement and excision of the mass; in more extensive or destructive cases, however, valve replacement has been performed. Recently, an alternative technique was reported by Wehman et al., consisting of a minimal incision and drainage, followed by closure and obliteration of the CCMA cavity [47].

However, as for MAC, patients with CCMA represent a high surgical risk population, since surgical valve replacement or repair is challenging because of older age, concomitant comorbidities, complex anatomy and technical difficulties [48]. Depending on the localization and anatomical features of CCMA, as well as its specific complications, various percutaneous approaches could potentially be taken into account as alternative solutions to surgical treatment.

Transcatheter mitral valve replacement (TMVR) using balloon-expandable aortic transcatheter heart valves (THVs), through percutaneous or hybrid approaches, has rapidly emerged as a feasible treatment option in high surgical-risk MAC patients, but data on the use of these interventions for the treatment of CCMA complicated by mitral regurgitation are lacking. Moreover, a lower 5-year survival rate has recently been demonstrated in patients undergoing valve-in-MAC (ViMAC) procedures [49]. Consequently, since CCMA represents a MAC variant, we can speculate that the poor long-term outcomes of ViMAC procedures also apply to this condition. Furthermore, the anatomic characteristics of CCMA, with its soft and fragile tissues, could render this strategy unsuitable.

Finally, with regard to the potential role of percutaneous transcatheter edge-to-edge repair (TEER), to the best of our knowledge, only one case of successful TEER intervention has hitherto been reported, concerning an 82-year-old woman suffering from CCMA, complicated with severe mitral regurgitation [50], judged unsuitable for surgery. Thus, further data are needed before this approach can be recommended.

In conclusion, the exact identification of CCMA complications is essential to plan the best patient-tailored treatment: the rupture of the mitral annulus with pseudoaneurysm formation or ventricular-atrial fistulization, as well as leakage of caseous material, needs surgical approach, as percutaneous treatment can be ineffective or detrimental. In contrast, significant MV dysfunction, i.e., mitral regurgitation at the level of leaflets, can be effectively approached by TEER or, alternatively, TMVR if annular calcification is extensive.

**Figure 6:**
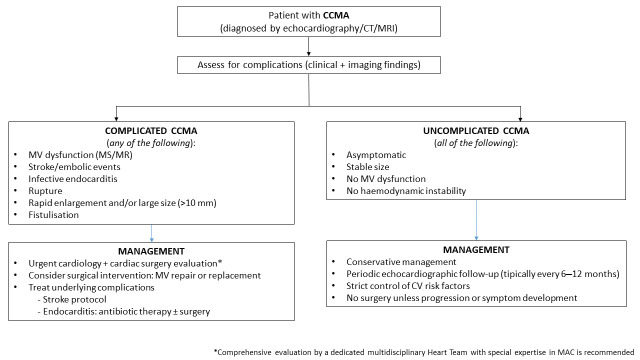
Proposed management of uncomplicated and complicated CCMA.

Based on such considerations, although the paucity of published data prevents us from deriving definitive indications about management, we are tempting to propose a general flow-chart strategy for CCMA, indicating potential indications for a conservative management or surgical/percutaneous treatment [Figure 6].

## 6. GAPS in Knowledge and Future Directions

Several questions about CCMA still remain open. Large prospective studies are needed not only to confirm the timing and indications for surgical resection of ruptured CCMA, but also to verify whether patients with high risk for systemic embolization and CCMA rupture may gain prognostic advantage from early treatment. Similarly, the timing of careful follow-up and surveillance has not been determined yet, as well as the exact characteristics of CCMA that should be considered at high risk for rupture. Moreover, it is not known whether patients with CCMA may benefit from endocarditis prophylaxis, similarly to other organic valve disease. On the other hand, pharmacologic treatment of embolization from CCMA is still unknown, because debris of caseous material does not regress with anti-coagulation. Finally, translational research about biological players, responsible for dynamic changes occurring inside MAC, is needed to address specific biomarkers and therapeutic targets. Preventive lipid-lowering therapy with anti-inflammatory effects, like statins, might reduce the evolution into CCMA of MAC or might reduce the dimension of CCMA, as described for atherosclerotic plaques. In the era of precision medicine, a tailored treatment for fragile patients might advocate a percutaneous approach to mitral valve dysfunction associated with CCMA, but large clinical studies are needed to verify whether transcatheter mitral valve prosthesis are beneficial for stabilization of CCMA.

## 7. Conclusions

CCMA is a relatively rare condition, often underdiagnosed due to its mainly asymptomatic presentation and limited awareness among imagers. However, the rupture of CCMA leads to local and systemic adverse events, the management of which is still under debate. Multimodality imaging is essential to distinguish stable MAC from uncomplicated and ruptured CCMA. In the absence of a standardized approach to ruptured CCMA, decisions regarding follow-up and treatment strategies are primarily based on the ability to identify high-risk clinical and imaging features.

## Figures and Tables

**Table 1:** Summary of published reports on complicated CCMA.

Study	Article Type	Type of CCMA and Related Complications	Dimensions	Imaging Modalities	Triggers/Predisposing Factors	Clinics	Management
Locorotondo G et al. [9]	Case report	Rupture of MV medial commissure and posterior annulus towards P3 and P2	N/A	TTE, TOE, CT, PET	Sepsis by Staphylococcus hominis	Multiple splanchnic and cerebral embolization, ventricular-atrial fistulization with severe MR	Surgical MV replacement with bioprosthetic valve
Pressman GS et al. [10] (*n* = 22)	Retrospective study	Vegetation located on the calcium deposits	N/A	TTE, TOE	Infectious by Staphylococcus Aureus	Endocarditis on native MV	N/A
Eicher JC et al. [11] (*n* = 15)	Retrospective study	Vegetations originating from a calcified mitral annulus	N/A	TTE, TOE	Diabetes mellitus, cancers, bacterial sepsis	Febrile coma, meningoencephalitis (53%); ring abscess, para-annular ventricular-atrial leakage	Conservative; 53% in-hospital mortality
Karakus A et al. [12]	Case report	A large mass on the posterior mitral annulus extending to the most basal area	30 × 26 mm	TTE, TOE	Hypertension, female, older age	Heart failure due to severe MR	Surgical MV replacement
Pozsonyi Z et al. [13]	Case report	A mass in the inferior and inferolateral mitral annulus, localized partially in the left ventricular wall, deforming the annulus and the basal part of the posterior leaflet	38 × 26 mm	TTE, TOE, CMR, CT	Female	Heart failure due to severe MR	Surgical MV replacement
Allwood RP et al. [14]	Case report	Partial rupture of the caseous calcification capsule wall including mobile remnants at the point of rupture	6 × 5 mm plus a 6 mm echogenic strand	TTE, TOE	Hypertension, female, older age	None	Conservative
Tanaka Y et al. [15]	Case report	Enlarging CCMA	31 × 18 mm	TTE, TOE, CT, CMR	Hypertension, female, older age	None	Conservative
Fujiwara M et al. [16] (*n* = 3)	Case series	Rupture/fistulous communication between the CCMA mass and the left ventricle, resulting in a mobile calcified structure	−10 × 9 mm −20 mm −29 × 14 mm	TTE, TOE	Hypertension, diabetes mellitus, and end-stage renal disease on hemodialysis	Cerebral embolization	Surgical MV repair/replacement
Chevalier B et al. [17]	Case report	CCMA with a mobile mass and an extension of approximately 5 cm toward the inferior wall of the left ventricle confirmed a small direct communication into the lumen of the left ventricle	34 × 16 mm	TTE, TOE, CT	Female, older age	Cerebral embolization	Surgical MV repair
Hamasaki A et al. [18]	Case report	A mobile, echo-dense mass with calcified capsule, dense and fine calcifications with fibrous tissue at the posterior mitral leaflet with 3 mm diameter defect	20 × 15 mm	TTE, CT	Female, diabetes mellitus and end-stage renal disease on hemodialysis	Cerebral embolization	Surgical MV repair
Gupta S et al. [19]	Case report	Ovoid cavity at the inferolateral mitral annulus, communicating with the left atrium, suggesting CCMA with rupture into the left atrium, plus another communication between this cavity and the left ventricle seen with color-Doppler flow jet directed into the lesion in systole	40 × 30 mm	TTE, TOE, CMR, CT	Female, hypertrophic cardiomyopathy, prior myocardial infarction, hypertension and transient ischemic attack	Heart failure due to severe MR	Surgical MV repair/replacement
Restivo A et al. [20]	Case report	A necrotizing anechoic cavity proximal to CCMA, invading the inferior-septal and inferior-basal wall of the LV, with blood flow communication between the LV and the cavity through a narrow neck (2 mm), with systolic inflow and diastolic swirling	35 × 40 mm	TTE, CT	Female, older age, hypertension and end-stage renal disease on hemodialysis, amyloidotic cardiomyopathy, severe aortic stenosis, recent pneumonia	Heart failure	Conservative
Mizuno H et al. [21]	Case report	Spherical calcified and ruptured mass at the center of the posterior mitral annulus, with a severe mitral regurgitation jet passed through the CCMA fistula. Soft string-like vegetation on the surface of the CCMA	15 mm	TTE, TEE	Sepsis by Streptococcus agalactiae	Multiple organ failure due to systemic embolism	Conservative—intra-hospital death

CMR = cardiac computed tomography; CT = computed tomography; MR = mitral regurgitation; MV = mitral valve; PET = positron emission tomography; TOE = transoesophageal echocardiography; TTE = transthoracic echocardiography.

**Table 2:** Multimodality imaging features of different forms of mitral annulus calcification and their management.

	MAC	CCMA	Ruptured CCMA
Imaging Features			
Echocardiography	Circumferential or focal echogenic mass with acoustic shadowing, but absence of an echo-lucent core	Large, spherical, echo-dense mass in the peri-annular mitral region, with no acoustic shadowing and central areas of echolucencies resembling liquefaction	Dense echogenic capsule with Echo-lucent center and mobile strands or superimposed echogenic soft tissue
Cardiac Magnetic Resonance	Hypointense in T1-weighted, T2-weighted, gradient echo, and steady-state free precession sequences	Heterogeneous, mainly hyperintense and partially hypointense center, with a hypointense rim on T1-weighted sequences Hypointense on T2-weighted short tau inversion recovery (STIR) sequences Decreased T1 and T2 relaxation times on mapping	Hypointense rim on T1-weighted and T2-weighted sequences, with black-blood filled cavity in spin-echo sequences and bright-blood filled cavity in gradient-echo and steady-state free precession sequences
Computed Tomography	Hyperdense mass with no central contrast filled space and no enhancement	Ovoid mass at the mitral annulus with a hypodense or hyperdense center and a peripheral rim of calcification with no enhancement (400 Hounsfield units)	A contrast filled space between the posterior mitral valve and basal lateral myocardial wall with a peripheral rim of calcification
Positron Emission Tomography	Lack of tracer uptake due to metabolically inactive or stable forms of calcification	Increased uptake of Fluorine-18-labeled fluorodeoxyglucose (18F-FDG) due to active inflammation Increased uptake of Gallium-citrate 67 (^67^Ga-citrate) and technetium Tc 99m methylene diphosphonate (99mTc-MDP) due to exuberant osteogenic activity	Mitral annulus uptake may be masked by intense and diffuse myocardial uptake secondary to the septic state
Clinical consequences	Mitral valve dysfunction (stenosis, regurgitation)	Mitral valve dysfunction (stenosis, regurgitation), atrial-ventricular blocks due to conductive system compression	Heart failure due to ventricular-atrial fistulization, myocardial dysfunction due to infiltration
Therapeutic implications	Risk of embolization secondary to atrial fibrillation due to annular dysfunction → anticoagulation indicated	Control of risk factors for atherosclerosis and atrial fibrillation	Risk of embolization of caseous material → anticoagulation ineffective
	Valve-in-MAC affected by poor outcome	Conservative strategy in asymptomatic patients	Surgical/percutaneous mitral valve replacement/repair in severe mitral valve dysfunction and/or caseous material embolization
	No size cut-off recommended for treatment	No size cut-off recommended for treatment	Surgical treatment may be indicated in large size mass (>10 mm) o rapidly increasing dimensions
	Endocarditis prophylaxis not indicated	Endocarditis prophylaxis useful	Endocarditis treatment indicated

## Data Availability

No new data were created or analyzed in this study. Data sharing is not applicable to this article.

## Abbreviations

The following abbreviations are used in this manuscript:

CCMA	Caseous calcification of mitral annulus
CMR	Cardiac magnetic resonance
CT	Computed tomography
MAC	Mitral annulus calcification
MV	Mitral valve
PET	Positron emission tomography
TOE	Transesophageal echocardiography
TTE	Transthoracic echocardiography
